# SOCS7/HuR/FOXM1 signaling axis inhibited high-grade serous ovarian carcinoma progression

**DOI:** 10.1186/s13046-022-02395-1

**Published:** 2022-05-27

**Authors:** Yanhua Du, Xiang Xu, Siang Lv, Han Liu, Hong Sun, Jun Wu

**Affiliations:** 1 Department of Gynecology, Obstetrics and Gynecology Hospital of Fudan University, Shanghai, 200011 P. R. China; 2 Department of Laboratory Medicine, Shanghai General Hospital Jiading Branch, Shanghai Jiao Tong University School of Medicine, Shanghai, 201803 P. R. China; 3 Department of Pathology, The Affiliated Hospital of Youjiang Medical University for Nationalities, Baise, 533000 P. R. China

**Keywords:** SOCS7, HuR, FOXM1, High-grade serous ovarian carcinoma, Prognosis

## Abstract

**Background:**

High-grade serous ovarian carcinoma (HGSOC) is clinically dominant and accounts for ~ 80% deaths in all types of ovarian cancer. The delayed diagnosis, rapid development, and wide dissemination of HGSOC collectively contribute to its high mortality rate and poor prognosis in the patients. Suppressors of cytokine signaling 7 (SOCS7) can regulate cytokine signaling and participate in cell cycle arrest and regulation of cell proliferation, which might also be involved in carcinogenesis. Here, we designated to investigate the functions and mechanisms of SOCS7 in HGSOC.

**Methods:**

The clinical correlation between SOCS7 and HGSOC was examined by both bioinformatics and analysis of tissue samples in patients. Gain/Loss-of-function examinations were carried out to assess the effectiveness of SOCS7 in cell viability, cell cycle, and tumor growth of HGSOC. Furthermore, the underlying mechanisms were explored by identifying the downstream proteins and their interactions via proteomics analysis and immunoprecipitation.

**Results:**

The expression of SOCS7, which was decreased in HGSOC tissues, was correlated with the clinical pathologic characteristics and overall survival of HGSOC patients. SOCS7 acted as a HGSOC suppressor by inhibiting cancer cell viability and tumor growth in vivo. The anti-HGSOC mechanism involves SOCS7’s regulatory effect on HuR by mediating its ubiquitination, the regulation of FOXM1 mRNA by HuR, as well as the interplays among these three clinically relevant factors.

**Conclusions:**

The SOCS7 correlates with HGSOC and suppresses its tumorigenesis through regulating HuR and FOXM1, which also suggests that SOCS7 is a prospective biomarker for the clinical management of ovarian cancer, especially HGSOC.

**Supplementary Information:**

The online version contains supplementary material available at 10.1186/s13046-022-02395-1.

## Background

Ovarian cancer among the leading causes of cancer-related mortality in females in the world, with an estimated annual diagnosis of more than 200,000 cases and more than 150,000 deaths [1]. The delayed onset symptoms, lack of diagnostic methods in the early stage, and tumor recurrence after chemotherapies all contribute to the high mortality rate and poor clinical management of patients with ovarian cancer [2, 3]. The type 1 neoplasms include clear, mucinous, transitional cell, and low-grade serous ovarian carcinoma, which are typically developed and progressed in a step-wise manner [4]. Whereas, high-grade serous ovarian carcinoma (HGSOC), as the type 2 neoplasm, is rapidly developed and widely disseminated, which is associated with the poorest overall prognosis among patients with all types of ovarian cancer [4, 5]. In fact, the aggressive HGSOC is the dominant subtype of clinically diagnosed ovarian cancer, and nearly 80% of the deaths caused by all types of ovarian cancer are attributed to HGSOC [5, 6]. Moreover, HGSOC is rarely diagnosed at its early stage, and compared with all the other subtypes of ovarian cancer, it possesses extensively higher cell proliferation rate, lower rate of programmed cell death, and extremely rapid cell invasiveness and metastasis [7, 8]. Understanding the molecular pathogenesis of HGSOC and developing efficient diagnostic/prognostic biomarkers are crucial steps for improving the long-term survival outcomes in patients with HGSOC by facilitating early diagnosis and proper and effective clinical management of the patients [6, 9].

Suppressors of cytokine signaling (SOCS) is a class of proteins that involve in the regulation of signal transduction [10, 11]. SOCS proteins can facilitate the proteasome-mediated degradation of activated cytokine-receptor complex, and therefore, further inhibit specific cytokine signaling and reduce the cell sensitivity towards other types of cytokines or even hormones [12, 13]. The SOCS family is consisted of eight protein members [14], among which, SOCS7 has been implicated in the regulation of downstream pathways of cytokines and the receptors of growth factors [15]. Meanwhile, SOCS7 may also possess an intriguing role in regulating cell cycle and tumor suppression [16]. It was reported that SOCS7 facilitates the nuclear translocation and accumulation of non-catalytic tyrosine kinase adaptor protein, which further induces DNA damage and cell cycle arrest [17]. At the same time, SOCS7 is also able to directly interact with growth factor receptor-bound protein, subsequently regulating cellular proliferation and differentiation [18, 19]. Previously, it was detected that SOCS7 is differentially expressed in benign and malignant breast tissues and has critical regulatory functions in insulin-like growth factor I- and hepatocyte growth factor-induced breast cancer [20, 21], indicating its involvement in the pathogenesis of human cancers. However, the function of SOCS7 in the pathogenesis of ovarian cancer, especially in HGSOC, is still unknown.

Therefore, we designated to investigate the relationship between SOCS7 and HGSOC, the roles of SOCS7 in inhibiting HGSOC, as well as the underlying molecular mechanisms. Through correlation analyses based on bioinformatics and patient cohorts, we demonstrated that SOCS7 expression was correlated with the prognosis of HGSOC patients. Further, by establishing SOCS7-knockdown and SOCS7-overexpression cellular models of HGSOC and analyzing the interactions between its downstream proteins, we revealed the anti-HGSOC effects of SOCS7 and illustrated the underlying molecular mechanism. This study establishes the foundation for the development of SOCS7 as potential biomarker for HGSOC.

## Materials and methods

### Bioinformatics analyses

The gene expression data were obtained from The Cancer Genome Atlas (TCGA) dataset for ovarian serous cystadenocarcinoma. Pathways that were substantially enriched in both SOCS7 high- and low- expression groups were identified by Gene set enrichment analysis (GSEA). The survival data were downloaded from the Kaplan Meier plotter website (http://www.kmplot.com/), using the best available cut-off value, including the GSE9891 (probe ID: 214015_at) and GSE26193 (probe ID: 226572_at) datasets for HGSOC [Grades 2 and 3; International Federation of Gynecology and Obstetrics (FIGO) stages III and IV].

### Patient information

Two cohorts of patients with HGSOC (Grades 2 and 3; FIGO stages III and IV) who underwent surgery in Obstetrics and Gynecology Hospital of Fudan University from 2012 to 2021 were included for the study. None of these patients had received chemotherapy or radiotherapy before surgery. Cohort 1 includes fresh 30 tumor and paired normal tissues and cohort 2 includes paraffin-embedded 68 tumor and 15 normal tissues. Approval of this study was obtained from the Ethics Committee of Fudan University.

### Immunohistochemistry (IHC) evaluation

Paraffin-embedded HGSOC tissue sections in cohort 2 were used for IHC studies. The IHC staining analysis was performed using anti-SOCS7 (bs-20151R), anti-HuR (bs-2651R), or anti-FOXM1 (bs-2687R; all from Bioss Antibodies Inc. Woburn, MA, USA) primary antibodies, followed by secondary antibody incubation (D-3004; Shanghai Long Island Biotechnology). Immunoreactivity was scored using the H-score system based on percentage of positively stained cells (0, < 5%; 1, 5%-25%; 2, 25%-50%; 3, 50%-75%; 4, > 75%) and staining intensity (0, negative; 1, weak; 2, moderate; 3, strong), which ranged from 0–12. HGSOC patients were divided into low expression (H-score < 4) or high-expression (H-score ≥ 4) group.

### Immunofluorescence microscopy

HGSOC cell lines were fixed by using 4% formaldehyde and then permeabilized by using 0.5% Triton X-100 in PBS. Subsequently, they were blocked for 30 min by using 1% bovine serum albumin in PBS, and the cells were further incubated with anti-SOCS7 (ab224589; Abcam), anti-HuR (ab200342; Abcam), Alexa Fluor 555-labeled Donkey Anti-Mouse IgG (H + L) (A0460, Beyotime Biotechnology, Nanjing, China), or Alexa Fluor 488-labeled Goat Anti-Rabbit IgG (H + L) (A0423, Beyotime Biotechnology) antibody before nuclear staining with DAPI (C1002, Beyotime Biotechnology). The stained cells were visualized and examined by using laser scanning confocal microscopy (Leica Microsystems Inc., Buffalo Grove, IL, USA).

### Cell culture

Human normal ovarian epithelial cell line IOSE80 and human HGSOC cell lines SNU119, CAOV3, OVCAR3, COV318, and OVCAR4 were obtained from the American Type Culture Collection (ATCC, Rockville, MD, USA) and cultured in 10% fetal bovine serum-supplemented RPMI-1640 medium in the presence of Penicillin–Streptomycin Solution (100X; SolarbioScience & Technology Co., Ltd., Shanghai, China) in 5% CO_2_ at 37 °C.

### Cell transfection

SOCS7 and HuR shRNA and scrambled shRNA lentivirus were purchased from Applied Biological Materials Company (Vancouver, Canada). Lentivirus production and purification were conducted as we previously described [22]. Full-length coding sequences of human HuR or FOXM1 genes were cloned into the expression vector pLVX-Puro (Addgen, Cambridge, MA, USA) for the construction of HuR or FOXM1 overexpression plasmids. Blank pLVX-Puro vector served as a negative control. Transfection was carried out based on Lipofectamine 2000 (Invitrogen, Carlsbad, CA, USA) following the protocols from the manufacturer (Life Technologies Corporation, Carlsbad, CA, USA). Following the transfection, the HGSOC cells were incubated for 48 h and subsequently transduced with the recombined expression vectors.

### Cell viability assay

Approximately 3 × 10^3^ CAOV3, OVCAR3, or COV318 cells/well were seeded in separate 96-well plates and then transduced with indicated lentiviral plasmids. Cell viability was measured at different time points (0, 24, 48, and 72 h) post-transduction, by using CCK-8 (cell counting kit-8; SAB Biotech, College Park, MD, USA). The absorbance at 450 nm was measured using a microplate reader.

### Analysis of cell cycle

Approximately 3 × 10^5^ CAOV3, OVCAR3, or COV318 cells/well were seeded in separate 6-well plates and then transduced with indicated lentiviral plasmids. At 48 h post-transduction, the cells were collected and incubated with 1 mg/ml RNase A (Sigma) and 50 μg/ml propidium iodide (Sigma) for 30 min at 25 °C for cell cycle analysis. Subsequently, the fluorescence intensities were determined by Accuri C6 flow cytometry (BD Biosciences, San Diego, CA, USA).

### Quantitative real-time polymerase chain reaction (qRT-PCR)

Total RNA was isolated from HGSOC tissues and cell lines by TRIzol reagent (Life Technologies, Grand Island, NY, USA), and reversely transcribed into cDNA by following the First Strand cDNA Synthesis Protocols (New England Biolabs, Ipswich, MA, USA). SYBR Green PCR Master Mix (Agilent Technologies, Santa Clara, CA, USA) was used for carrying out qRT-PCR, based on an ABI7300 System (Applied Biosystems, Carlsbad, CA, USA). The following primers were used for qRT-PCR: GAPDH-F:5ʹ-AATCCCATCACCATCTTC-3ʹ, GAPDH-R:5ʹ-AGGCTGTTGTCATACTTC-3ʹ; SOCS7-F:5ʹ-GGTTTGTGGCTTCCTGATG-3ʹ, SOCS7-R:5ʹ-GTGGGCTGTGTTTATGGTG-3ʹ; HuR-F:5ʹ-TCTGCGCTTGGCCTTAGTC-3ʹ, HuR-R:5ʹ-AACCGTCTTCGGGTGCTTC-3ʹ; FOXM1-F:5ʹ-AATGGCAAGGTCTCCTTC-3ʹ, FOXM1-R:5ʹ-AGCAGTGGCTTCATCTTC-3ʹ. The 2^−ΔΔCT^ method was applied for determining the fold changes of target genes at the transcript level.

### Western blot assay

Total protein was isolated and collected from HGSOC cell lines with radioimmunoprecipitation assay lysis buffer (Santa Cruz Biotech, Santa Cruz, CA, USA). The isolated proteins with equal quantity were further separated by sodium dodecyl sulphate–polyacrylamide gel electrophoresis (SDS-PAGE; 10% or 12%), followed by transferring onto separate polyvinylidene fluoride membranes (Beijing Solarbio Science& Technology, Beijing, China). The antibodies used for immunoblotting were the following: GAPDH (Cell Signaling Technology, Beverly, MA, USA), SOCS7 (ab224589; Abcam, Cambridge, MA, USA), HuR (ab238528; Abcam), Cyclin D1 (ab16663; Abcam), Survivin (ab76424; Abcam), CDC25B (ab124819; Abcam), and FOXM1 (ab207298; Abcam). After incubating with horseradish peroxidase-conjugated secondary antibody (Beyotime Institute of Biotechnology), the signals were further examined based on an enhanced chemiluminescence system (Bio-Rad, Richmond, CA, USA).

### Immunoprecipitation and liquid chromatography/mass spectrometry (LC/MS) analyses

Full-length coding sequence of human SOCS7 was cloned into a pCMV-FLAG vector. The 293 T cells expressing FLAG-tagged SOCS7 were constructed by transfection with Lipofectamine 2000 (Invitrogen) according to the instructions from the manufacturer and lysed with lysis buffer (1% Triton X-100, 150 mM NaCl, 20 mM Tris pH7.5, and 1 mM EDTA) supplemented with protease inhibitor cocktail (Sigma-Aldrich, MO, USA). The cell lysates were then incubated with anti-FLAG beads (Sigma-Aldrich) overnight incubated at 4 °C. After washing with lysis buffer five times, the immunoprecipitated protein complex was eluted by using FLAG peptide (Sigma-Aldrich) for 1 h at 4 °C. Protein samples were then resolved by SDS-PAGE for Coomassie Blue staining. Protein bands were excised from the gel and subjected to in-gel digestion with trypsin, and the resulting peptides were dissolved in a solution comprising 0.1% trifluoroacetic acid and 2% acetonitrile for analysis with Orbitrap Fusion mass spectrometer (Thermo Fisher Scientific, San Jose, CA) coupled with Easy nLC (Thermo Fisher Scientific, Waltham, MA, USA) as previously described. The raw mass files generated by the Orbitrap Fusion instrument were processed using Proteome Discoverer software (Thermo Scientific, version 1.4) integrated with the MASCOT (Matrix Science, London, UK; version 2.3.2) search engine for protein identification. Data were searched against the Human UniProtKB/Swiss-Prot database. Proteins and peptides with a false discovery rate less than 1% were selected.

### Co-immunoprecipitation (co-IP) and ubiquitination assay

Cell lysates were prepared with lysis buffer (1% Triton X-100, 150 mM NaCl, 20 mM Tris pH7.5, and 1 mM EDTA) supplemented with protease inhibitor cocktail (Sigma-Aldrich), incubated with normal IgG (ab172730; Abcam), anti-HuR (ab200342; Abcam), or anti-SOCS7 (ab224589; Abcam) antibody, followed by further incubating with Protein A/G PLUS-Agarose beads (sc-2003; Santa Cruz Biotechnology) at 4 °C for 2 h. Then, the immunocomplex was three-time washed by the lysis buffer for Western blot analysis with antibodies against SOCS7 (ab224589; Abcam), HuR (ab200342; Abcam), and ubiquitin (ab7780; Abcam).

### RNA immunoprecipitation (RIP) assay

Magna RNA immunoprecipitation (RIP) RNA-Binding Protein Immunoprecipitation kit (Millipore) was used for the RIP assay following the manufacturer’s instructions. Cells transduced with indicated lentiviral plasmids were prepared using RIP lysis buffer, the lysed cells were centrifuged at 12,000 × g for 10 min at 4˚C, and the RNA–protein complexes were incubated with anti-HuR (ab200342; Abcam) or anti-IgG antibody (ab172730; Abcam) at 4 °C for 1 h. Once incubation was complete, agarose beads and 50 µl of protein A/G were added and cells were incubated for a further 60 min at 4˚C. Subsequently, the precipitated beads were washed with RIP-wash buffer for 10 min at 4 °C and then RIP-lysis buffer for 5 min at 4 °C. The RNA in the immunoprecipitated complex and the RNA in the previously saved input fraction were released by incubating cells at 65˚C for 2 h with 200 mM NaCl and 20 µg proteinase K, which reversed the cross-linking. The amount of FOXM1 mRNA bound by HuR was determined by qRT-PCR.

### Plasmid construction and dual-luciferase reporter assay

The wide-type and mutant FOXM1 3' UTR reporter plasmid was constructed by cloning PCR-amplified sequences from the 3' UTR of FOXM1 cDNA into a pGL3-basic firefly luciferase reporter plasmid (Promega, Madison, WI, USA). CAOV3 cells transduced with HuR-expressing vector were seeded into 24-well plates and transfected with 400 ng of FOXM1 3' UTR reporter plasmid (WT or Mutant). To normalize the transfection efficiency, cells were cotransfected with 50 ng of pRL-TK containing renilla luciferase. After 48 h, cells were washed with PBS and lysed using passive lysis buffer. Luciferase activity was assessed using a Dual-Luciferase Reporter Assay system (Promega) following the manufacturer’s instructions.

### Measurement of mRNA stability

Cells transduced with the indicated lentiviral plasmids were incubated with 5 μg/ml transcription inhibitor actinomycin D (Sigma, St. Louis, MO, USA). Total RNA was isolated at time intervals of 0, 2, 4 and 6 h following actinomycin D addition. FOXM1 mRNA was determined using qRT-PCR, and the relative amount of FOXM1 mRNA at 0 h following actinomycin D treatment was set to 100%.

### Tumor xenografts in animals

CAOV3 cells stably expressing pLVX-Puro-SOCS7 or blank pLVX-Puro were subcutaneously injected into male nude mice (6-week-old; *n* = 6 per group). At the 33^rd^ day post-inoculation, tumors were collected, weighed, photographed, and analyzed by qRT-PCR, Western blot and immunofluorescence staining by using anti-Ki67 antibody (ab15580; Abcam). All the animal studies were approved by the Ethics Committee of Fudan University.

### Statistical analyses

Data were presented in the form of ‘mean ± standard deviation’, and validated by three independent experiments or multiple independent mice trials, as indicated. Experimental results were analyzed with GraphPad Prism Software Version 8.4.2 (GraphPad Software Inc., La Jolla, CA, USA). Two-tailed Student's t-test and analysis of variance were applied for two groups comparisons and multiple groups comparisons, respectively. Wilcoxon test and nonparametric Kruskal–Wallis test were both utilized for evaluating the differences of tumor tissues and controls. Pearson's or Spearman's correlation was used for measuring association between expression levels of SOCS7/FOXM1, FOXM1/HuR or SOCS7/HuR. A statistical significance was considered when the *P* value was less than 0.05.

## Results

### SOCS7 correlates with the prognosis of HGSOC patients

**Fig. 1:**
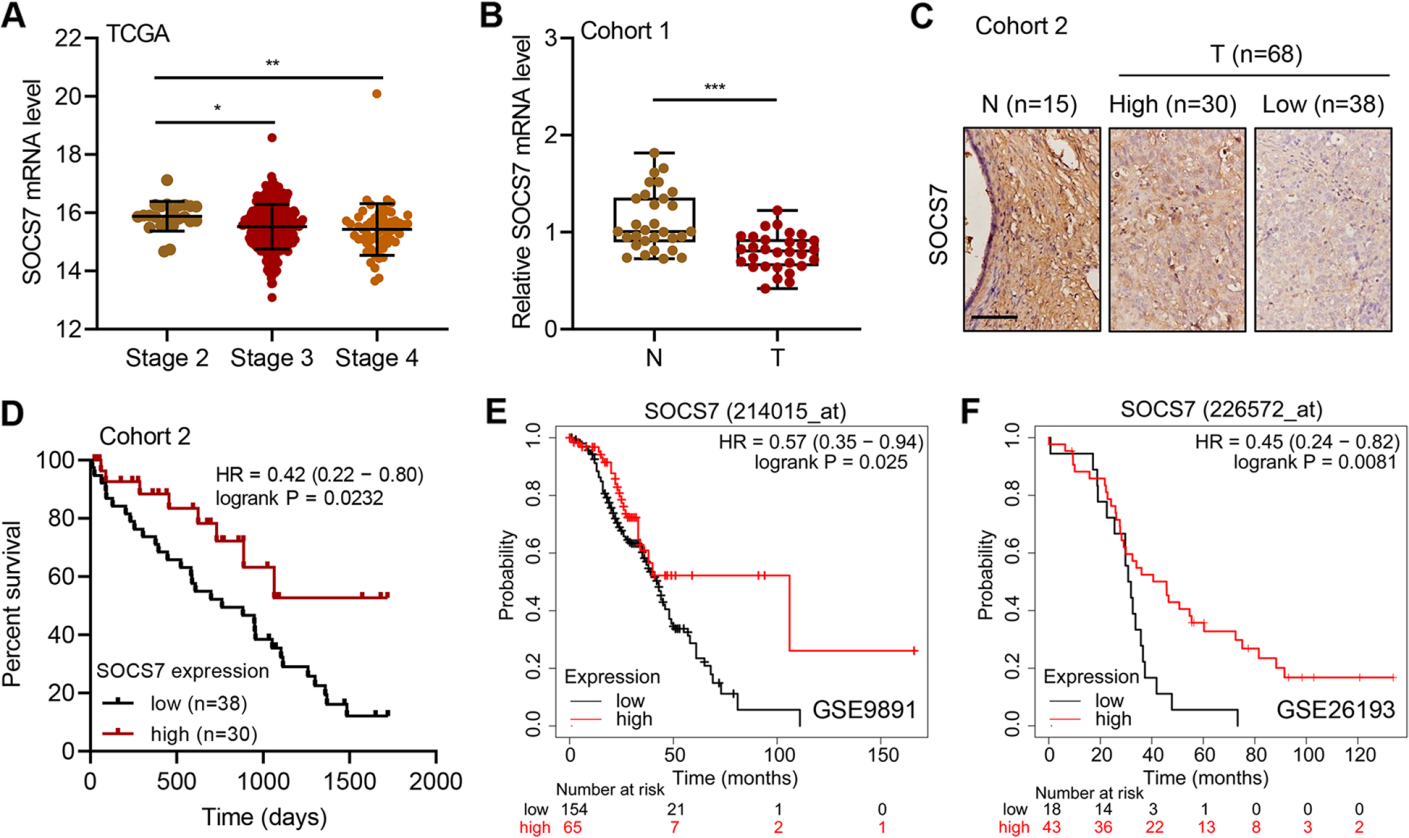
SOCS7 expression is associated with HGSOC progression and survival rate of HGSOC patients. **a** and **b** SOCS7 mRNA levels in different stages of ovarian serous cystadenocarcinoma based on TCGA database (**a**) and ovarian tumors and paired normal tissues from 30 HGSOC patients in cohort 1 (**b**). **c** IHC staining and analysis for SOCS7 protein expression in 68 ovarian tumors and 15 normal tissues from HGSOC patients in cohort 2. Scale bar = 100 μm. **d-f** The overall survival of HGSOC patients in cohort 2 (**d**), the GSE9891 dataset (**e**), and the GSE26193 dataset (**f**). *P < 0.05, **P < 0.01, and ***P < 0.001

In order to examine the correlation between SOCS7 and HGSOC, we first analyzed the expression level of SOCS7 in HGSOC patients. Based on the dataset from TCGA, we found that the transcript levels of SOCS7 in HGSOC patients with tumor stages 3 (*P* < 0.01) and 4 (*P* < 0.05) were significantly lower than that in the patients with tumor stage 2 (Fig. 1a). Meanwhile, based on the analysis of tissues collected from patient cohort 1, we observed that the transcript level of SOCS7 in the tumor tissues from the HGSOC patients was significantly (*P* < 0.001) lower than that in the paired normal tissues (Fig. 1b).

To further investigate the protein expression of SOCS7 in HGSOC patients, we divided the collected tissues from HGSOC patients in cohort 2 into two groups according to their SOCS7 protein levels, based on the IHC staining results (Fig. 1c). The high expression of SOCS7 was significantly (*P* = 0.0232) correlated with high survival rate of HGSOC patients in cohort 2 (Fig. 1d). Similarly, based on the datasets from GSE9891 (*P* = 0.025; Fig. 1e) and GSE26193 (*P* = 0.0081; Fig. 1f), we also found the significantly positive correlation between the SOCS7 mRNA level and overall survival rate of HGSOC patients. Furthermore, the expression of SOCS7 was notably correlated with two of the clinicopathologic characteristics, FIGO stage (*P* = 0.006) and grade (*P* = 0.003) of tumor, in the patients with HGSOC in cohort 2 (Table 1). Univariate analysis revealed the significant correlation between overall survival of HGSOC patients and FIGO stage (*P* = 0.002), grade (*P* = 0.009), and SOCS7 level (*P* < 0.0001), while multivariate analysis revealed their independent correlations in cohort 2 (*P* = 0.012, *P* = 0.045, and *P* = 0.002, respectively; Table 2).

**Table 1:** Correlation between the SOCS7 expression and clinicopathological characteristics in 68 patients with HGSOC

Characteristics	No. Patients (*n* = 68)	SOCS7 expression		*P* value
		Low No. (%)	High No. (%)	
Age (years)				0.357
> 57	36	22 (61.1%)	14 (38.9%)	
≤ 57	32	16 (50.0%)	16 (50.0%)	
FIGO stage				0.006
III	53	25 (47.2%)	28 (52.8%)	
IV	15	13 (86.7%)	2 (13.3%)	
Grade				0.003
2	12	2 (16.7%)	10 (83.3%)	
3	56	36 (64.3%)	20 (35.7%)

**Table 2:** Univariate and multivariate Cox regression analysis of overall survival in 68 patients with HGSOC

Variables	Univariate analysis		Multivariate analysis	
	HR (95% CI)	*P* value	HR (95% CI)	*P* value
Age (> 57 vs. ≤ 57)	1.69 (0.95–3.14)	0.073	1.46 (0.83–2.64)	0.194
Grade (3 vs. 2)	0.41 (0.26–0.68)	0.002	0.48 (0.30–0.84)	0.012
FIGO stage (IV vs. III)	0.26 (0.07–0.78)	0.009	0.41 (0.14–0.98)	0.045
SOCS7 level (low vs. high)	0.25 (0.12–0.48)	< 0.0001	0.42 (0.22–0.74)	0.002

### SOCS7 regulates cell cycle, inhibits cell viability, and suppresses tumor growth in HGSOC cell lines and xenografts

**Fig. 2:**
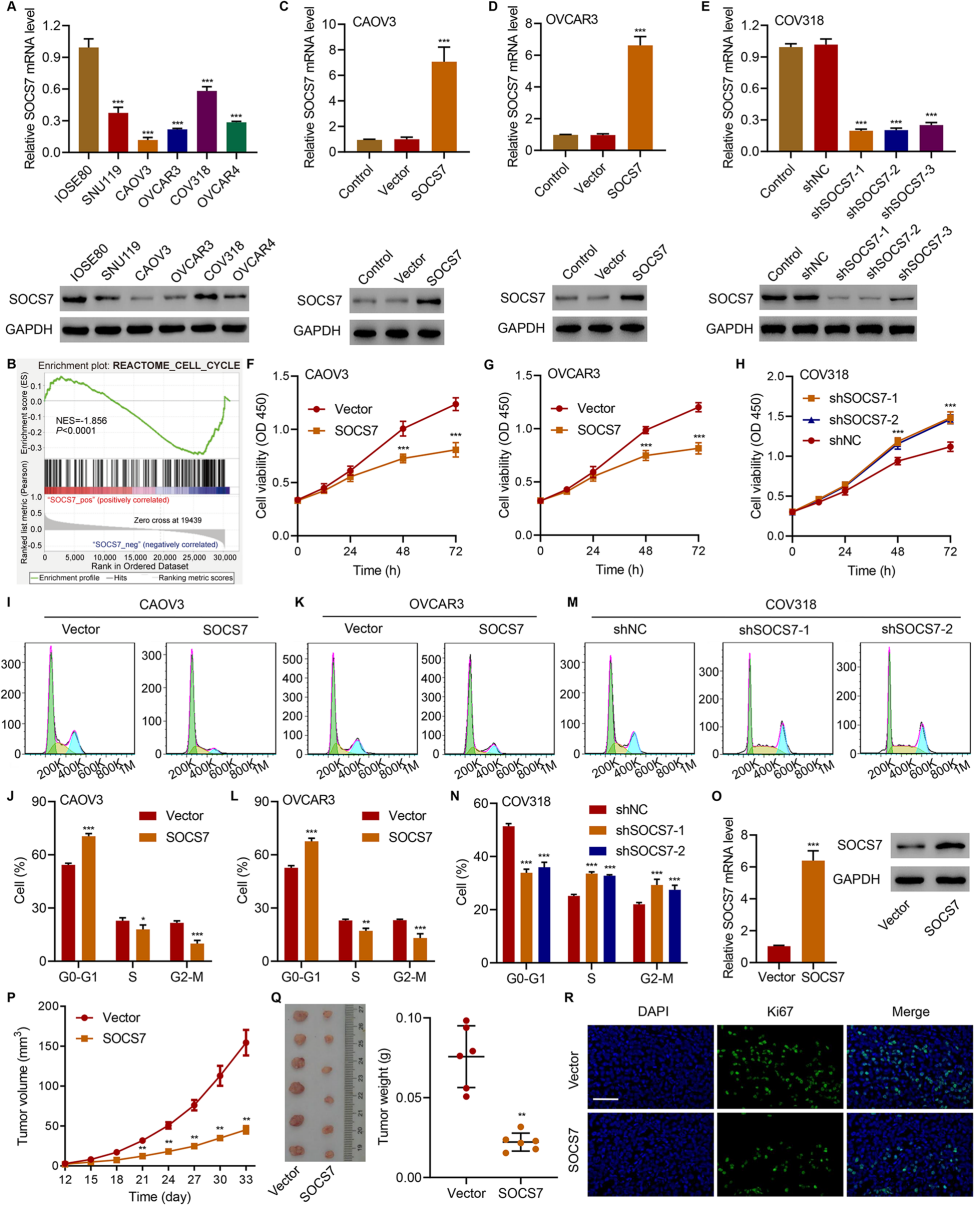
SOCS7 regulates HGSOC cell growth both in vitro and in vivo. **a** The mRNA and protein levels of SOCS7 in various HGSOC cell lines and human ovarian epithelial cell line IOSE80. **b** Association between SOCS7 expression and cell cycle, based on GSEA analysis. **c-e** SOCS7 overexpression and knockdown in HGSOC cells transduced with indicated lentiviral vectors. **f**–**h** Viability and **i-n** cell cycle of HGSOC cells transduced with indicated lentiviral vectors. **o-r** CAOV3 cells transduced with pLVX-Puro-SOCS7 or blank pLVX-Puro were injected into nude mice, and SOCS7 expression levels (**o**), tumor volume (**p**), weight (**q**), and the immunofluorescence staining for Ki67 (**r**) were determined. Scale bar = 50 μm. *P < 0.05, **P < 0.01, and ***P < 0.001, in related with IOSE80, shNC or Vector

The mRNA and protein levels of SOCS7 were compared among IOSE80 cells and various HGSOC cell lines. We observed that all the examined HGSOC cells, including SNU119, CAOV3, OVCAR3, COV318, and OVCAR4 cells, contained significantly lower expression levels of SOCS7, compared with that in the IOSE80 cells (Fig. 2a). Moreover, the GSEA analysis revealed that SOCS7 expression was significantly (*P* < 0.0001) and negatively (NES = -1.856) correlated with the cell cycle pathway in HGSOC cells (Fig. 2b). Among the above mentioned HGSOC cell lines, the lowest levels of SOCS7 were found in CAOV3 and OVCAR3 cells, while the highest level was detected in COV318 cells (Fig. 2a). Therefore, we overexpressed SOCS7 in CAOV3 (Fig. 2c) and OVCAR3 (Fig. 2d) cells, and silenced SOCS7 in COV318 cells (Fig. 2e), to further investigate the roles of SOCS7 in HGSOC.

Substantial (*P* < 0.001) reduction of viability was observed in the CAOV3 (Fig. 2f) and OVCAR3 (Fig. 2g) cells overexpressing SOCS7 compared with control cells. On the contrary, SOCS7 knockdown in COV318 cells substantially (*P* < 0.001) increased their viability (Fig. 2h). Furthermore, SOCS7 overexpression in both CAOV3 (Fig. 2i and j) and OVCAR3 (Fig. 2k and l) cells substantially increased the percentage of cells in G0-G1 phase (*P* < 0.001), while decreased the proportions of cells in S (*P* < 0.05 for CAOV3; *P* < 0.01 for OVCAR3) and G2-M (*P* < 0.001) phases. In contrast, silencing SOCS7 expression in COV318 cells substantially (*P* < 0.001) reduced the percentage of cells in G0-G1 phase but elevated those of cells in S and G2-M phases (Fig. 1m and n). These results indicate the regulatory role of SOCS7 on HGSOC cell viability and cell cycle.

We next evaluated the effect of SOCS7 overexpression on HGSOC tumor growth in vivo. We observed that SOCS7 overexpression significantly increased the expression of SOCS7 in tumor xenografts at day 33 (Fig. 2o) and that the volume of the developed tumor at days 21–33 (Fig. 2p) and the tumor weight at day 33 (Fig. 2q) in mice injected with SOCS7-overexpressing CAOV3 cells were substantially (*P* < 0.01) lower than those in the control mice. Additionally, Ki67-immunofluorescent staining revealed that SOCS7 overexpression inhibited cell proliferation in the developed tumors (Fig. 2r). These results suggest that SOCS7 can suppress HGSOC tumorigenicity.

### SOCS7 inhibits HGSOC cell viability by mediating the ubiquitination of HuR

**Fig. 3:**
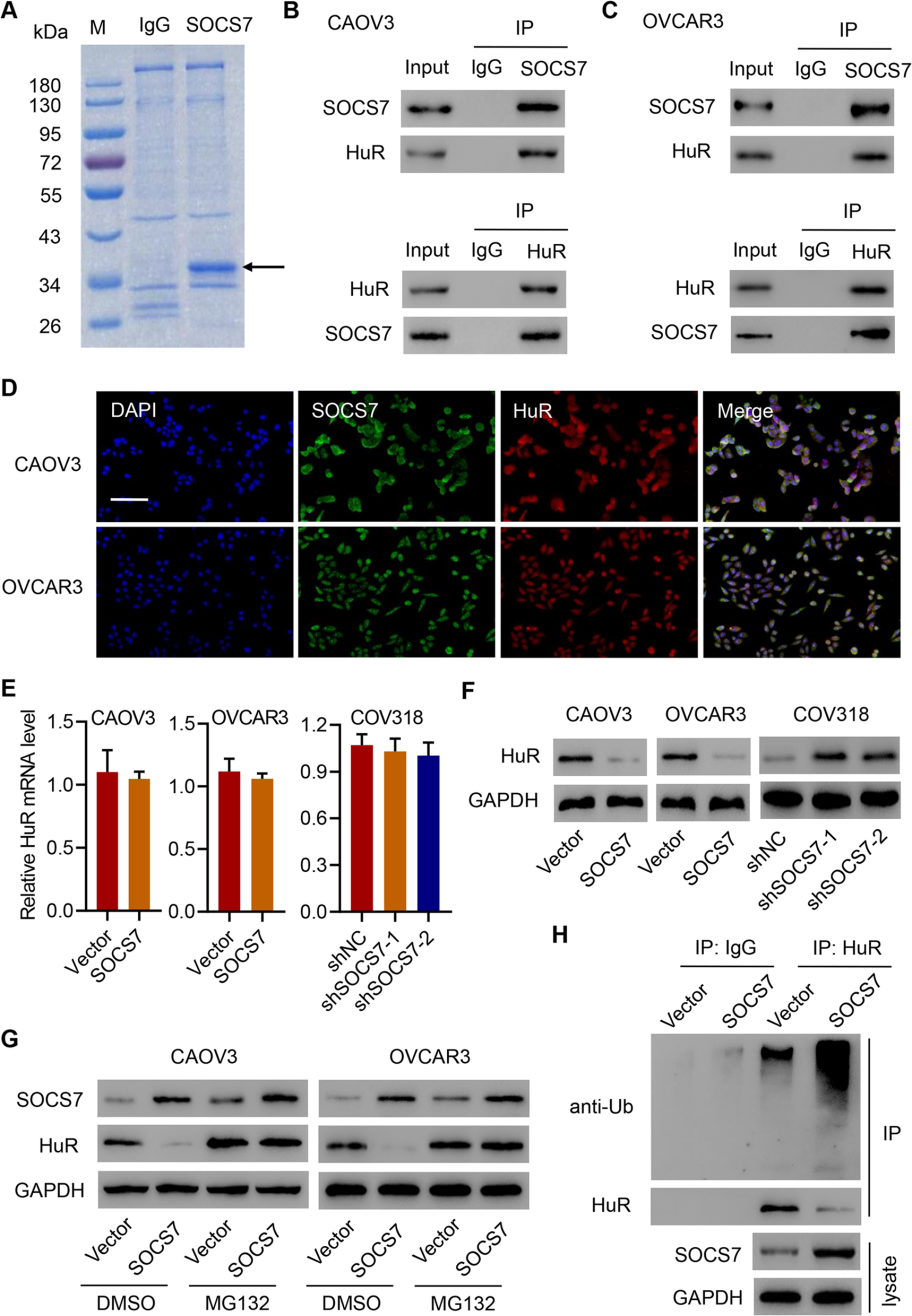
Interactions between HuR and SOCS7. **a** Purification of SOCS7 immunocomplex (arrows). Proteins were separated on SDS-PAGE and stained by Coomassie Blue. **b** and **c** The interactions between HuR and SOCS7 in CAOV3 (**b**) and OVCAR3 (**c**) cell lysates. **d** Subcellular localizations of SOCS7 (green) and HuR (red). Nuclei were stained with DAPI (blue) for reference. Scale bar = 50 μm. **e **and** f** The mRNA (**e**) and protein (**f**) levels of HuR in CAOV3, OVCAR3, and COV318 cells transduced with indicated lentiviral vectors. **g** SOCS7 and HuR protein expression in CAOV3 and OVCAR3 cells transduced with indicated lentiviral vectors and treated with 10 μM proteasome inhibitor (MG132) or DMSO. **h** SOCS7/HuR protein expression and the ubiquitination of HuR in CAOV3 cells

We further investigated the underlying mechanism of SOCS7’s anti-tumor effect; we performed a proteomics analysis to identify candidate proteins associated with SOCS7 and found that HuR was one of the top-ranked proteins (Fig. 3a and Supplementary Tab. 1). Based on our co-IP analysis, SOCS7 interacted with HuR protein in both CAOV3 (Fig. 3b) and OVCAR3 (Fig. 3c) cells. In addition, immunofluorescence staining showed that both SOCS7 and HuR could be localized to the nucleus and the cytoplasm in CAOV3 and OVCAR3 cells (Fig. 3d). Based on the results from co-IP and immunofluorescence staining, the interaction and co-localization of SOCS7 and HuR were confirmed. Furthermore, we found that neither SOCS7 overexpression nor its knockdown could influence the transcript level of HuR (Fig. 3e), whereas the expression level of HuR protein was downregulated in SOCS7-overexpressing CAOV3 and OVCAR3 cells but upregulated in COV318 cells with SOCS7 knockdown (Fig. 3f). However, proteasome inhibitor MG132 treatment could rescue the reduced protein level of HuR in SOCS7-overexpressing CAOV3 and OVCAR3 cells (Fig. 3g). In addition, the overexpression of SOCS7 in CAOV3 cells also promoted the ubiquitination of HuR (Fig. 3h).

**Fig. 4:**
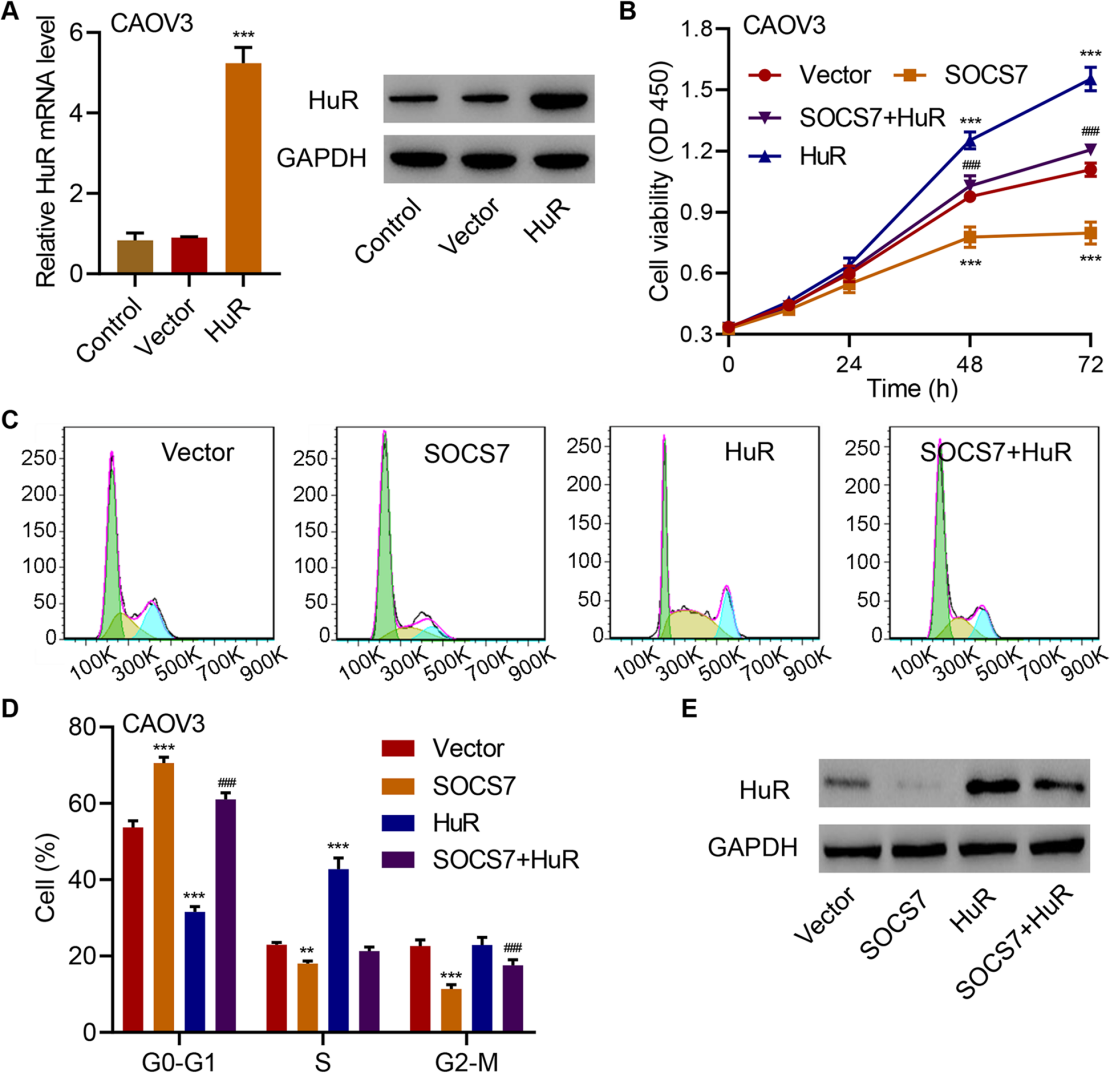
HuR is involved in the regulation of HGSOC cell viability and cell cycle progression by SOCS7. **a** HuR overexpression in CAOV3 cells. **b** Viability, **c** and **d** cell cycle distribution of CAOV3 cells transduced with indicated lentiviral vectors. **e** HuR protein expression in CAOV3 cells transduced with indicated lentiviral vectors. **P < 0.01, ***P < 0.001, in related with Vector. ^###^*P* < 0.001, in related with SOCS7

We further examined the function of HuR in HGSOC to investigate the mechanism underlying the tumor-inhibitory effect of SOCS7. We overexpressed HuR in CAOV3 cells (Fig. 4a), and found that HuR overexpression could considerably (*P* < 0.001) increase CAOV3 cell viability (Fig. 4b), decrease the percentage of cells in G0-G1 phase (Fig. 4c and d), and increase the percentage of cells in S phase (Fig. 4c and d). Moreover, the overexpression of HuR in CAOV3 cells considerably counteracted the effects of SOCS7 overexpression on CAOV3 cell viability (Fig. 4b), cell cycle (Fig. 4c and d) and HuR expression (Fig. 4e), indicating the critical role of HuR in the HGSOC-suppressive function of SOCS7.

### FOXM1, which is regulated by HuR, contributes to the anti-HGSOC function of SOCS7

**Fig. 5:**
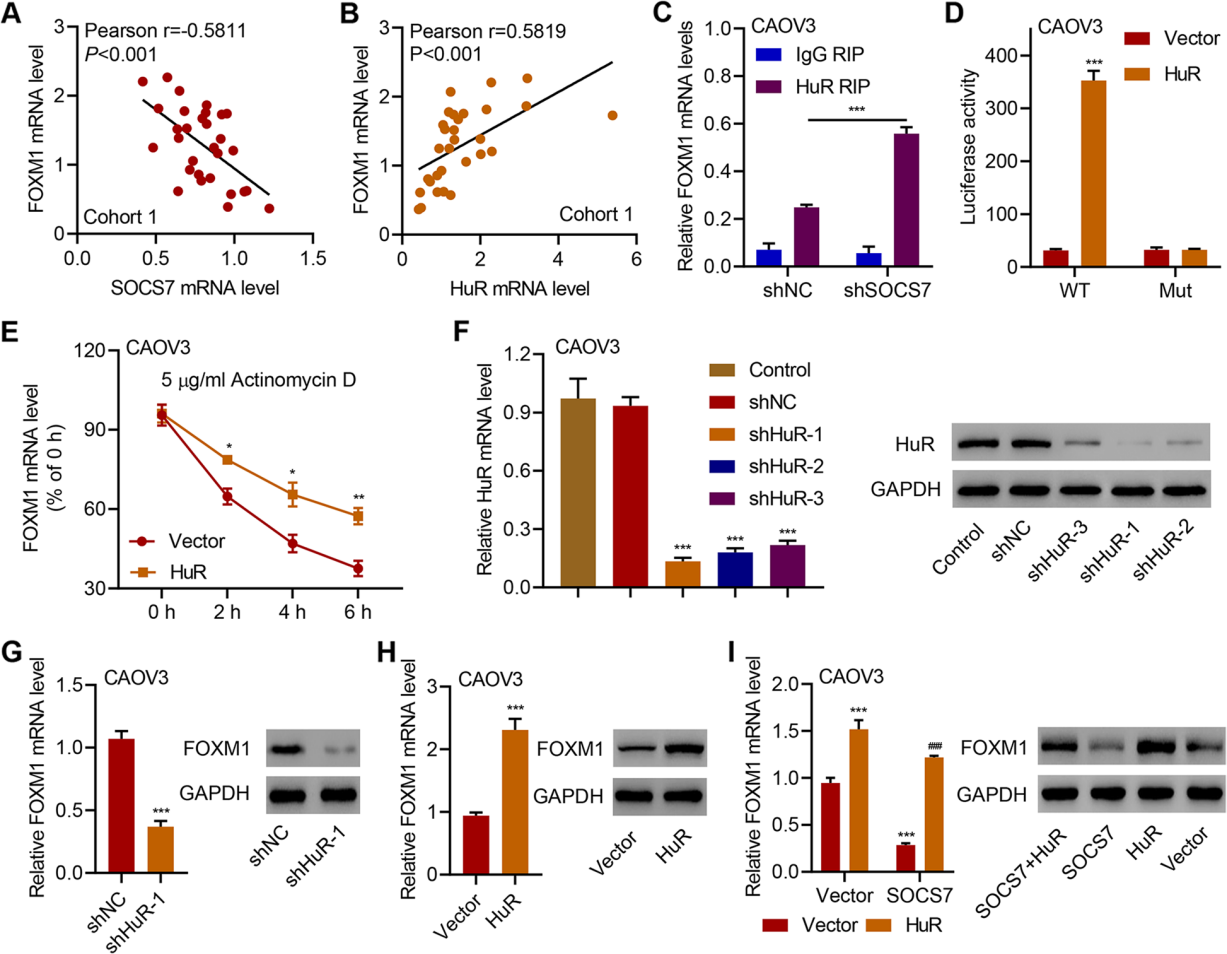
HuR binds to the 3′UTR of FOXM1 and regulates its transcription. **a** and **b** Pearson's correlation scatter plots for FOXM1-SOCS7 (**a**) and FOXM1-HuR (**b**) of HGSOC patients (*n* = 30) in cohort 1. **c** Additional HuR RIPs followed by qRT-PCR on precipitates validated HuR binding of FOXM1 mRNA in CAOV3 cells transduced with indicated lentiviral vectors. **d** Relative luciferase activity of FOXM1 3'UTR in CAOV3 cells transduced with indicated lentiviral vectors. **e** Relative mRNA levels of FOXM1 in CAOV3 cells transduced with indicated lentiviral vectors and treated with 5 μg/ml actinomycin D. **f-i** HuR and FOXM1 expression in CAOV3 cells transduced with indicated lentiviral vectors. *P < 0.05, **P < 0.01 and ***P < 0.001, in related with shNC, Vector. ^###^*P* < 0.001, in related with SOCS7

Forkhead box M1 (FOXM1) is a transcription factor and a target of HuR [23], and overexpression and activation of FOXM1 is frequent and associated with worse prognosis in patients with HGSOC [24, 25]. Moreover, FOXM1 overexpression also contributes to cell proliferation, cell cycle progression, chemoresistance, migration, and invasion in HGSOC [25–27]. Based on Pearson's correlation analysis, we demonstrated the significantly (*P* < 0.001) negative (*r* = -0.5811) correlation between SOCS7 and FOXM1 mRNA levels (Fig. 5a) as well as the significantly (*P* < 0.001) positive (*r* = 0.5819) correlation between HuR and FOXM1 mRNA levels (Fig. 5b). Moreover, SOCS7 overexpression in both CAOV3 and OVCAR3 cells inhibited the expression levels of FOXM1 downstream target genes Cyclin D1, Survivin and CDC25B (Supplementary Fig. 1a). In contrast, silencing SOCS7 in COV318 cells promoted expression levels of Cyclin D1, Survivin and CDC25B (Supplementary Fig. 1b). As shown in Supplementary Fig. 1c, the expression levels of FOXM1 and HuR in tumor xenografts were also decreased after SOCS7 overexpression. Subsequent qRT-PCR analyses of additional HuR-RIPs confirmed HuR binding of FOXM1 mRNA, and this interaction was increased in CAOV3 cells with SOCS7 silencing (Fig. 5c). HuR is known to bind the untranslated regions (UTRs) of mRNAs, which have major roles in post-transcriptional regulation We found that the overexpression of HuR significantly (*P* < 0.001) increased the activity of wild-type FOXM1 3′UTR, but not that of mutant FOXM1 3′UTR in CAOV3 cells (Fig. 5d). Accumulating evidence indicates that HuR controls mRNA activity by regulating mRNA stability. To examine the influence of transcription, the transcription inhibitor actinomycin D was used. As shown in Fig. 5e, the half-life of FOXM1 mRNA in CAOV3 cells transduced with HuR-expressing vector was much longer than that in CAOV3 cells transduced with blank vector. These results clearly demonstrate that HuR stabilizes FOXM1 mRNA, which plays an important role in the regulation of FOXM1 gene expression. To verify these results, we further knocked down HuR expression in CAOV3 cells (Fig. 5f). The mRNA and protein levels of FOXM1 in CAOV3 cells were significantly (*P* < 0.001) downregulated by the knockdown of HuR (Fig. 5g) and upregulated by the overexpression of HuR (Fig. 5h). Additionally, overexpression of HuR in SOCS7-overexpressing CAOV3 cells could significantly (*P* < 0.001) counteract the inhibitory effect of SOCS7 overexpression on FOXM1 at mRNA and protein levels (Fig. 5i).

**Fig. 6:**
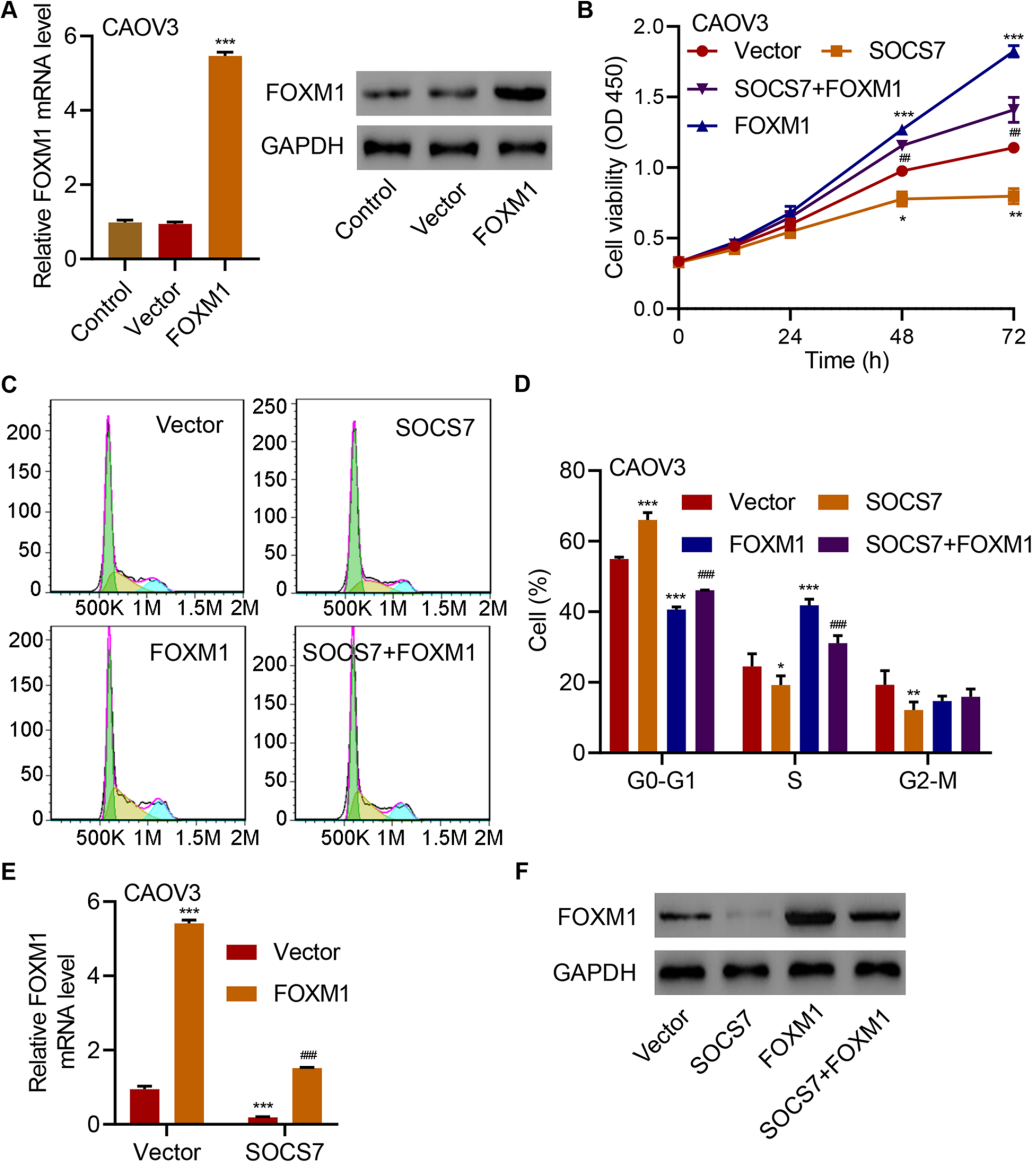
FOXM1 is involved in the regulation of HGSOC cell viability and cell cycle progression by SOCS7. **a** FOXM1 expression in CAOV3 cells transduced with indicated lentiviral vectors. **b** Viability, **c** and **d** cell cycle distribution of CAOV3 cells transduced with indicated lentiviral vectors. **e** and **f** FOXM1 mRNA and protein expression in CAOV3 cells transduced with indicated lentiviral vectors. *P < 0.05, **P < 0.01 and ***P < 0.001, in related with Vector. ^##^*P* < 0.01, and ^###^*P* < 0.001, in related with SOCS7

We further investigated the roles of FOXM1 in HGSOC through overexpressing FOXM1 in CAOV3 cells (Fig. 6a). We observed that FOXM1 overexpression could considerably promote CAOV3 cell viability (*P* < 0.001 at 48 and 72 h; Fig. 6b), reduce the percentage of cells in G0-G1 phase (*P* < 0.001; Fig. 6c and d), and increase the proportions of cells in S (*P* < 0.05) and G2-M phases (*P* < 0.01; Fig. 6c and d). Moreover, FOXM1 overexpression in CAOV3 cells considerably counteracted the regulatory effects of SOCS7 overexpression on cell viability (*P* < 0.01 at 48 and 72 h; Fig. 6b), cell cycle (*P* < 0.001; Fig. 4c and d), and the cellular mRNA and protein levels of FOXM1 (Fig. 6e and f), demonstrating the significance of FOXM1 in the anti-HGSOC functions of SOCS7.

### The expression levels of SOCS7, HuR, and FOXM1 are related to each other and correlated with prognosis in HGSOC patients

**Fig. 7:**
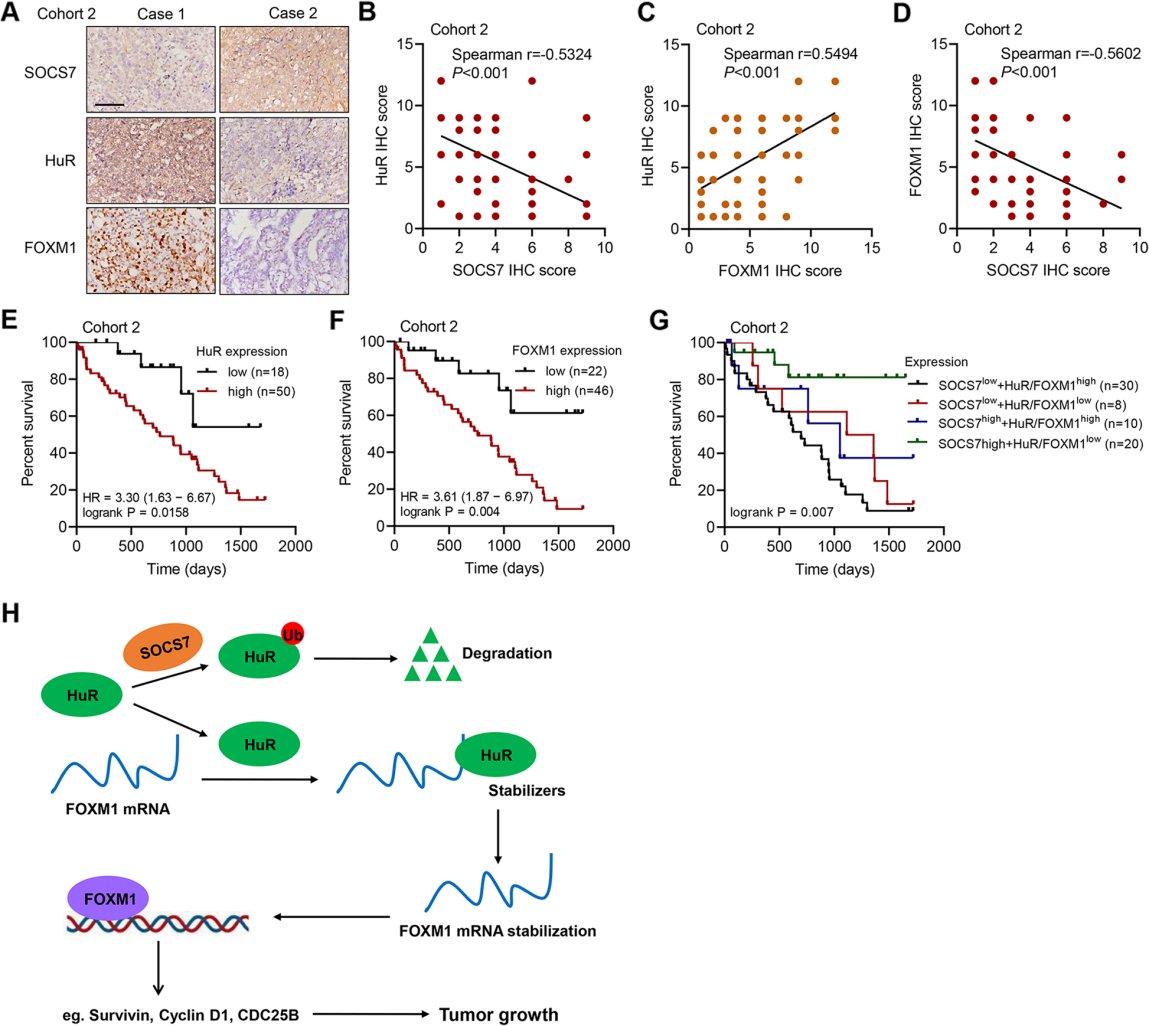
The relationship among the expression levels of SOCS7/HuR/FOXM1 in the tumor tissues of HGSOC patient and their correlations with survival rate of HGSOC patients. **a** IHC staining analysis of SOCS7, HuR, and FOXM1 in the tumor tissues of HGSOC patients in cohort 2. Scale bar = 100 μm. **b**-**d** Spearman's correlation scatter plots for HuR-SOCS7 (**b**), HuR-FOXM1 (c) and FOXM1-SOCS7 (**d**) of HGSOC patients in cohort 2. **e** and **f** The overall survival of HGSOC patients with different protein levels of HuR (**e**) or FOXM1 (**f**) in cohort 2. **g** The overall survival of HGSOC patients with different protein levels of SOCS7, HuR, and FOXM1 in cohort 2. **h** Schematic model of the regulation of HGSOC tumor growth by SOCS7/HuR/FOXM1

We further performed IHC staining on tumor tissues of HGSOC patients from cohort 2 (Fig. 7a). The Spearman's correlation analysis revealed that SOCS7 expression was significantly (*P* < 0.001) and negatively (*r* = -0.5324) correlated with HuR expression (Fig. 7b), the expression of HuR was significantly (*P* < 0.001) and positively (*r* = 0.5494) correlated with the expression of FOXM1 (Fig. 7c), and the expression of SOCS7 was significantly (*P* < 0.001) and negatively (*r* = -0.5602) correlated with the expression of FOXM1 (Fig. 7d) in cohort 2. These results indicate that the expression levels of these three proteins are related to each other in HGSOC patients. Furthermore, significantly negative correlation was observed between the percent survival rate of HGSOC patients and the expression level of HuR (*P* = 0.0158; Fig. 7e) or FOXM1 (*P* = 0.004; Fig. 7f) in cohort 2. Significant differences in the overall survival rates were also identified among the HGSOC patients with different protein levels of SOCS7, HuR, and FOXM1 in cohort 2. As expected, the patients with higher expression of HuR + FOXM1 and lower expression of SOCS7 (SOCS7^low^ + HuR/FOXM1^high^) had the shortest survival time, while the patients with lower expression of HuR + FOXM1 and higher expression of SOCS7 (SOCS7^high^ + HuR/FOXM1^low^) had the longest survival time (Fig. 7g). From these observations, we conclude that decreased expression levels of SOCS7 and elevated expression levels of HuR/FOXM1 may identify HGSOC patients with poor prognosis.

## Discussion

SOCS family proteins inhibit related signaling pathways and regulate multiple cytokine responses and growth factor signals [28]. The aberrant expression or activation of SOCS was correlated with the development and progression of various types of human tumors [29]. In the current study, we observed that one of the SOCS family members, SOCS7, was downregulated in the tumor tissues in HGSOC patients. This finding was supported by previous report suggesting that the transcript level of SOCS7 was inversely correlated with the tumor, node, and metastasis (TNM) stage of cancer [16]. Moreover, a recent study also illustrated that SOCS7 expression was connected with poor prognosis in ovarian cancer patients [30], based on which we revealed the noticeable correlation between the expression of SOCS7 protein and survival rate or clinicopathological features of HGSOC patients. Further, the results from univariate and multivariate analyses indicate the recognition of SOCS7 as a promising independent prognostic biomarker for HGSOC patients.

The upregulation of SOCS7 was found to restrict the aggressive cellular activities of prostate cancer [31]. Similarly, SOCS7 was also demonstrated to negatively control the growth factor signaling in breast cancer cells and limit their growth and migration [32]. Consistent with these observations, here we observed the inhibitory effect of SOCS7 on HGSOC cell viability, cell cycle progression, and tumor development. In addition, SOCS proteins can regulate STAT signaling and facilitate p53 activation, both of which contribute to the cell cycle arrest at the G1 phase [33], which might explain the finding in the present study that SOCS7 overexpression caused more HGSCO cells to accumulate at the G0-G1 phase.

The RNA-binding protein Hu antigen R (HuR) belongs to embryonic lethal abnormal vision-like protein family, which binds to adenine or uracil-enriched motifs to enhance mRNA stabilization and post-translational regulation or target genes [34, 35]. For the first time, we identified the association and interaction between SOCS7 and HuR proteins in the study. As a matter of fact, the aberrant or dysregulated post-translational modifications of HuR, especially the ubiquitin-proteasomal degradation, have been implicated in carcinogenesis processes [36, 37]. Since all SOCS proteins share a common conserved motif (SOCS box) that is recognized as the binding site for inducing ubiquitin-mediated proteasomal degradation of target substrates [38, 39], we proposed that SOCS7 might be able to mediate HuR degradation. We further confirmed that SOCS7 could facilitate the ubiquitination of HuR and down-regulate its cellular protein level. HuR has been reported to promote oncogenesis of multiple human cancers through its RNA-regulatory effect and its significance in cell cycle modulation, cell proliferation, and cell differentiation [36, 40, 41]. In this study, we have also revealed that HuR could facilitate HGSOC cell viability and regulate cell cycle. Meanwhile, HuR overexpression counteracted the tumor-suppressive effect of SOCS7, demonstrating the interplay between these two proteins in HGSOC pathogenesis. Collectively, SOCS7 constrains the tumorigenicity of HGSOC, potentially through mediating HuR ubiquitination and minimizing its cellular level in ovarian cancer.

FOXM1 is one of the transcriptional factors in the FOX family, and its transcriptional activation contributes to homologous recombination repair and G1-S/G2-M transitions in cell cycle [42, 43]. Previous study has suggested the proto-oncogenic roles of FOXM1 and its downstream target genes Cyclin D1, Survivin and CDC25B in tumors [44], which include facilitating cell cycle progression and inducing tumor cell proliferation, as well as its relation with the poor prognosis of cancer patients [45, 46]. Similarly, we also found that FOXM1 overexpression could stimulate HGSOC cell viability and regulate tumor cell cycle. The expression level of FOXM1 can be employed to predict the survival probability of HGSOC patients. Furthermore, the interaction between HuR and FOXM1 was also revealed by the current study, for which we demonstrated that HuR could stabilize FOXM1 mRNA by binding to FOXM1 3′UTR. This finding is in line with the well-known HuR activities of specifically binding to the targeted genes for enhancing their transcription [36, 47–49], and it also explains the oncogenic roles of HuR in HGSOC. Therefore, the expression levels of SOCS7, HuR, and FOXM1 are closely correlated with prognosis in HGSOC patients, and these three proteins participate in the pathogenicity and progression of HGSOC. By elucidating their interactions and regulatory functions, the overall suppressive role of SOCS7 in HGSOC was well explained.

## Conclusion

To summarize, SOCS7 is downregulated in HGSOC tumor, and its expression is associated with the clinicopathologic features and survival probability of HGSOC patients, demonstrating its correlation with HGSOC prognosis. Meanwhile, SOCS7 interacts with and mediates the ubiquitination of HuR, while HuR binds with FOXM1 3'UTR and enhances its mRNA stability. HuR and FOXM1 are both involved in enhancing HGSOC cell viability and promoting tumor growth (Fig. 7h). Through these above mentioned mechanisms, SOCS7 exhibits HGSOC-suppressive activities in reducing cell viability, regulating cell cycle, and restricting tumorigenicity. In addition, SOCS7, HuR, and FOXM1 are related with each other and independently correlated with the survival rate of HGSOC patients. These findings recognize SOCS7 as a HGSOC suppressor and reveal its anti-tumor mechanisms based on the regulatory SOCS7/HuR/FOXM1 axis, which suggests its potential in serving as a prognostic biomarker for HGSOC.

## Supplementary Information

**Additional file 1: Supplementary Table 1. **HuR/ELAV-like Protein 1 was identified to interact with SOCS7 via liquid chromatography/mass spectrometry (LC/MS).

**Additional file 2: Supplementary Figure 1. **Protein expression in HGSOC cell lines and in tumor xenografts. **a **and** b **Protein levels of Cyclin D1, Survivin and CDC25B in CAOV3 (**a**), OVCAR3 (**a**), and COV318 (**b**) cells transduced with indicated lentiviral vectors.** c** CAOV3 cells transduced with pLVX-Puro-SOCS7 or blank pLVX-Puro were injected into nude mice, and protein levels of HuR and FOXM1 in tumor xenografts were determined (one representative Western blot was chosen among different repeats).

## Data Availability

The datasets used and/or analyzed during the current study are available from the corresponding author on reasonable request.

## Abbreviations

HGSOC: High-grade serous ovarian carcinoma
SOCS7: Suppressors of cytokine signaling 7
HuR: RNA-binding protein Hu antigen R, ELAV-like Protein 1
FOXM1: Forkhead box M1
TNM: Tumor, node, and metastasis
TCGA: The Cancer Genome Atlas
FIGO: International Federation of Gynecology and Obstetrics
IHC: Immunohistochemistry
qRT-PCR: Quantitative real-time polymerase chain reaction
co-IP: Co-immunoprecipitation
RIP: RNA immunoprecipitation
