# Supplementary figures and images for: SOCS7/HuR/FOXM1 signaling axis inhibited high-grade serous ovarian carcinoma progression
Source: J Exp Clin Cancer Res. 2022 May 27;41:185. doi: 10.1186/s13046-022-02395-1 (PMC9137060; doi:10.1186/s13046-022-02395-1)

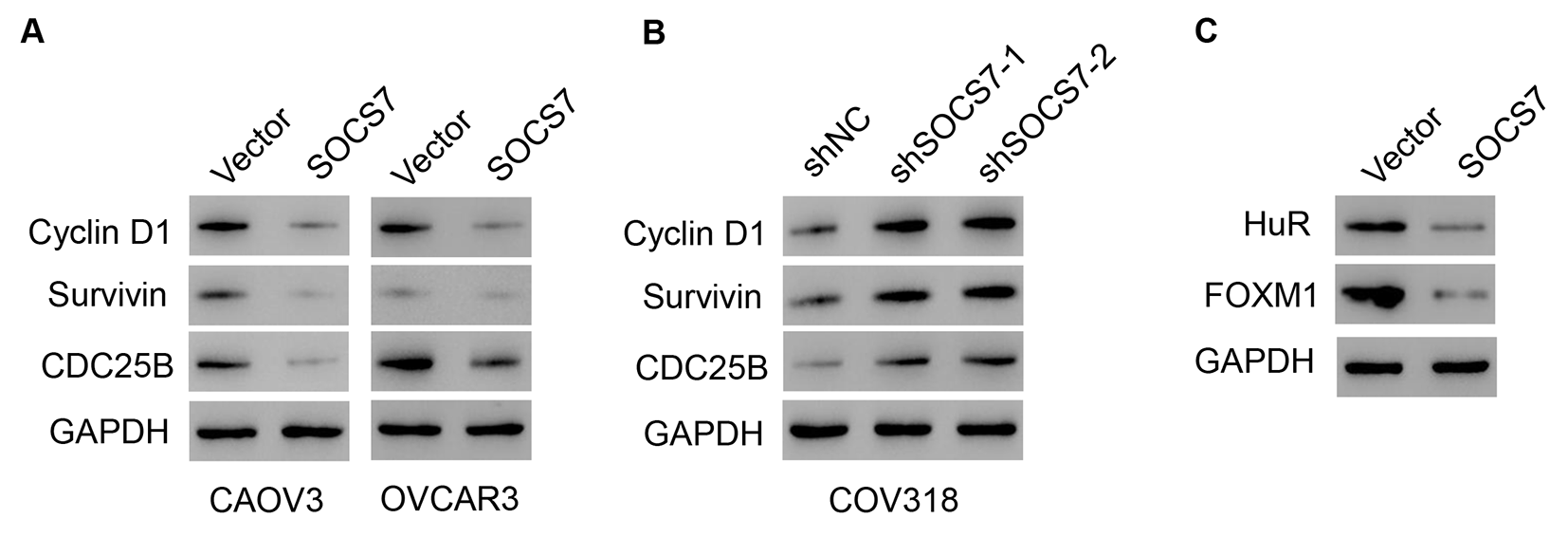

Supplement: Supplementary file 2 — Additional file 2: Supplementary Figure 1. Protein expression in HGSOC cell lines and in tumor xenografts. a and b Protein levels of Cyclin D1, Survivin and CDC25B in CAOV3 (a), OVCAR3 (a), and COV318 (b) cells transduced with indicated lentiviral vectors. c CAOV3 cells transduced with pLVX-Puro-SOCS7 or blank pLVX-Puro were injected into nude mice, and protein levels of HuR and FOXM1 in tumor xenografts were determined (one representative Western blot was chosen among different repeats). [file 13046_2022_2395_MOESM2_ESM.tif]
